# Implementation considerations when expanding health worker roles to include safe abortion care: a five-country case study synthesis

**DOI:** 10.1186/s12889-017-4764-z

**Published:** 2017-09-21

**Authors:** Claire Glenton, Annik M. Sorhaindo, Bela Ganatra, Simon Lewin

**Affiliations:** 1 Global Health Unit, Norwegian Institute of Public Health, Oslo, Norway; 2 Independent Consultant in Reproductive and Sexual Health, Mexico City, Mexico; 3 UNDP/UNFPA/UNICEF/WHO/World Bank Special Programme of Research, Development and Research Training in Human Reproduction (HRP), World Health Organization, Geneva, Switzerland; 4 Global Health Unit, Norwegian Institute of Public Health / Health Systems Research Unit, South African Medical Research Council, Cape Town, South Africa

**Keywords:** Reproductive health, Abortion, Health systems, Human resources for health, Task shifting, Role expansion, Implementation, Service delivery

## Abstract

**Background:**

Allowing a broader range of trained health workers to deliver services can be an important way of improving access to safe abortion care. However, the expansion of health worker roles may be challenging to implement. This study aimed to explore factors influencing the implementation of role expansion strategies for non-physician providers to include the delivery of abortion care.

**Methods:**

We conducted a multi-country case study synthesis in Bangladesh, Ethiopia, Nepal, South Africa and Uruguay, where the roles of non-physician providers have been formally expanded to include the provision of abortion care. We searched for documentation from each country related to non-physician providers, abortion care services and role expansion through general internet searches, Google Scholar and PubMed, and gathered feedback from 12 key informants. We carried out a thematic analysis of the data, drawing on categories from the SURE Framework of factors affecting the implementation of policy options.

**Results:**

Several factors appeared to affect the successful implementation of including non-physician providers to provide abortion care services. These included health workers’ knowledge about abortion legislation and services; and health workers’ willingness to provide abortion care. Health workers’ willingness appeared to be influenced by their personal views about abortion, the method of abortion and stage of pregnancy and their perceptions of their professional roles. While managers’ and co-workers’ attitudes towards the use of non-physician providers varied, the synthesis suggests that female clients focused less on the type of health worker and more on factors such as trust, privacy, cost, and closeness to home. Health systems factors also played a role, including workloads and incentives, training, supervision and support, supplies, referral systems, and monitoring and evaluation. Strategies used, with varying success, to address some of these issues in the study countries included values clarification workshops, health worker rotation, access to emotional support for health workers, the incorporation of abortion care services into pre-service curricula, and in-service training strategies.

**Conclusions:**

To increase the likelihood of success for role expansion strategies in the area of safe abortion, programme planners must consider how to ensure motivation, support and reasonable working conditions for affected health workers.

**Electronic supplementary material:**

The online version of this article (10.1186/s12889-017-4764-z) contains supplementary material, which is available to authorized users.

## Background

Allowing a broader range of trained health workers to deliver safe abortion care is one way of improving access to these services. However, the expansion of health worker roles in abortion care may be challenging to implement, at least initially. This paper presents the results of a multi-country case study synthesis where we explore factors that may influence the implementation of the expansion of health worker roles to include abortion care services in five countries: Bangladesh, Ethiopia, Nepal, South Africa, and Uruguay. We undertook this synthesis to inform the World Health Organization (WHO) guidelines on “Health worker roles in providing safe abortion care and post-abortion contraception” [1]. These guidelines provide evidence-based recommendations about the inclusion of a range of health workers in the delivery of abortion, post-abortion care and post-abortion contraception.

Ethiopia, Nepal, South Africa and Uruguay currently permit abortion in a broad range of circumstances [2–9]. While abortion law per se is restrictive in Bangladesh, menstrual regulation is defined as the evacuation of the uterus of a woman at risk of being pregnant to ensure a state of non-pregnancy [10], and is not regulated by the penal code restricting abortion [10].

In all five countries, abortion care services are provided as part of the formal healthcare system. In addition to care delivery by obstetricians, gynaecologists and non-specialist physicians, a variety of healthcare professionals provide these services, including midwives, nurses and auxiliary nurses. In Nepal and Ethiopia, governments also use lay or community health workers to perform certain supportive tasks related to abortion care such as community education and referral to health facilities [11, 12].

The increased use of non-physician providers to deliver healthcare services is typically regarded as a promising strategy for improving access to healthcare. However, such role expansion constitutes a complex intervention that has a number of implications for health workers, for women, and for the organisation of care. The objective of this study was to explore factors influencing the implementation at scale of non-physician provider role expansion to include the delivery of abortion care services.

## Methods

We conducted a multi-country case study synthesis based on data from five countries. The case-study approach involves analysing phenomena in ‘real life’ settings by using a range of different types of evidence. This approach can be particularly useful when the goal is to compare and explore processes within and across settings [13, 14].

### Country selection

Through consultation with experts, we selected countries with at-scale, national programmes that used non-physician providers to deliver some of their abortion care services; were located in Africa, Asia and South America; had been running for at least 5 years; and had a reasonable level of documentation available in English or Spanish. Non-physician providers can include a range of healthcare providers, including associate clinicians, midwives, auxiliary nurse midwives, nurses, auxiliary nurses and lay health workers. Given the resource-intensive nature of the related data collection and analysis, we limited ourselves to five countries: Bangladesh, where the menstrual regulation programme was established in 1979; and South Africa, Nepal, Ethiopia and Uruguay, where the current abortion programmes were established in 1996, 2002, 2005 and 2012 respectively.

### Data collection, analysis and synthesis

We searched for written documentation, including evaluation reports and academic study reports, about factors affecting abortion care delivery and role expansion in each country after the introduction of their national abortion programme. We searched using general internet searches; through Google Scholar and PubMed (see Additional file 1); and by searching the reference lists of included documents. Our search was carried out in May 2014 and repeated in November 2016. In addition, we purposively sampled 12 key informants from non-governmental and governmental organisations and research institutions who had been involved in the implementation and / or assessment of the abortion care programmes in the five countries. We invited these key informants to contribute to the case study synthesis in order to contribute to the WHO Guidelines on abortion care. We asked each key informant to give feedback to our preliminary analysis of the written reports, and to provide any additional written resources. In addition, we asked each key informant to clarify issues that were unclear in the written documentation and to offer additional factors they thought may have influenced the implementation of role expansion strategies in these programmes. We communicated with key informants through phone or skype meetings lasting from 30 min to one and a half hours and/or through email.

The conceptual framework of this study was based on the SURE (Supporting the Use of Research Evidence) framework. The framework, which provides a comprehensive list of possible factors that may influence the implementation of health system interventions [15] was used to develop our data extraction sheet and informed our analysis (see Table 1).

**Table 1:** Using the SURE Framework to identify factors affecting the implementation of role expansion for abortion care

Level	Factors affecting implementation of policy options included in the SURE Framework [15]	Factors affecting the implementation of role expansion for abortion care identified in this study
Recipients of care	Knowledge and skills	
	Attitudes regarding programme acceptability, appropriateness and credibility	Women’s attitudes to and experiences of different types of health workers
	Motivation to change or adopt new behaviour	
Providers of care	Knowledge and skills	Health workers’ knowledge about abortion legislation and services
	Attitudes regarding programme acceptability, appropriateness and credibility	Health workers’ willingness to provide abortion care Health workers’ understanding and use of conscientious objection
	Motivation to change or adopt new behaviour	
Other stakeholders	Knowledge and skills	
	Attitudes regarding programme acceptability, appropriateness and credibility	Co-workers’ attitudes towards role expansion
	Motivation to change or adopt new behaviour	
Health system constraints	Accessibility of care	
	Financial resources	
	Human resources	
	Educational and training system, including recruitment and selection	Health worker training
	Clinical supervision, support structures and guidelines	Health workers’ access to supervision and emotional support
	Internal communication	
	External communication	
	Allocation of authority	
	Accountability	Monitoring and evaluation of health workers
	Community participation	
	Management and/or leadership	
	Information systems	
	Scale of private sector care	
	Facilities	
	Patient flow processes	Health workers’ access to referral systems
	Procurement and distribution systems	Health workers’ access to supply chains
	Incentives	Health worker workloads and incentives
	Bureaucracy	
	Relationship with norms and standards	
Social and political constraints	Ideology	
	Governance	
	Short-term thinking	
	Contracts	
	Legislation or regulation	
	Donor policies	
	Influential people	
	Corruption	
	Political stability and commitment	

CG or AS read each of the included documents and extracted any data that described factors tied to the implementation of abortion care delivery as it relates to role expansion, and summarised these. CG and AS then read and re-read the data summaries, identified key themes, and discussed the definitions and boundaries of each emerging theme and how these themes related to the SURE framework. BG and SL commented on emerging themes. CG and AS also went through all feedback from key informants. We categorised similar themes that emerged from different countries together within the relevant SURE framework category.

## Results

**Fig. 1:**
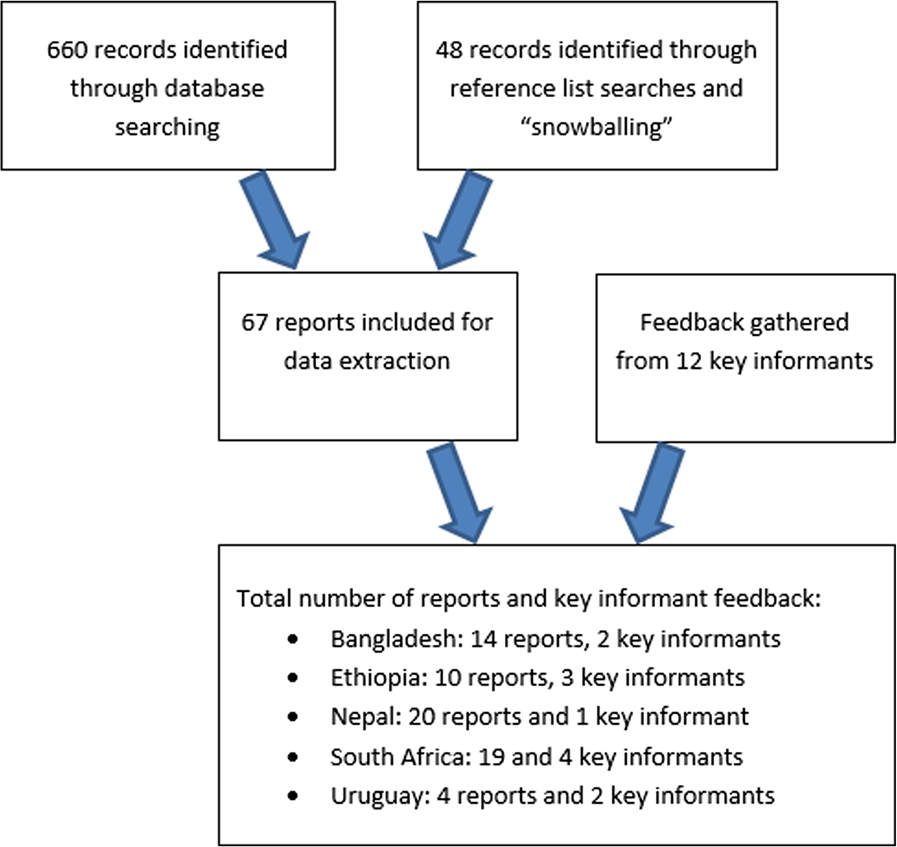
Flow diagram

We included and extracted data from 67 written reports and feedback from 12 key informants (see Fig. 1 Flow diagram; and Additional file 2 Overview of documents that contributed to the synthesis findings). Our final analysis was based on 14 reports and feedback from two key informants from Bangladesh, ten reports and feedback from three key informants from Ethiopia, 20 reports and feedback from one key informant from Nepal, 19 reports and feedback from four key informants from South Africa, and four reports and feedback from two key informants from Uruguay. While some of the reports were descriptions or opinion pieces, most were published academic study reports or programme evaluation reports, and most had used qualitative semi-structured interviews or quantitative surveys to collect data.

Very few of the reports focused on the topic of role expansion specifically, but dealt more generally with factors affecting the delivery of abortion care services. However, these factors are important to the success of any role expansion strategy and so are discussed below. All of the reports gave some information about factors affecting abortion care service delivery by non-physician providers. However, several of these reports combined this information with information about specialist and non-specialist doctors. In the results section, we have used the term “health workers” to refer to data from reports that deal with either non-physician providers only or non-physician and physician providers in combination.

We identified a number of factors that appeared to have influenced the implementation of non-physician provider role expansion for abortion care services in the five countries. Some of these factors were only mentioned briefly, including factors tied to health worker accreditation. In this paper we focus on factors that may have influenced health workers’ inclusion in the delivery of abortion care once legal and accreditation requirements in their settings had been fulfilled. These factors included willingness to provide abortion care; health workers’ knowledge about abortion legislation and services; managers’ and co-workers’ attitudes towards role expansion; women’s attitudes to and experiences of different types of health workers; and health systems factors, including workloads and incentives, health worker training and supervision, supply chains and referral systems, and monitoring and evaluation (see Table 1).

### Health workers’ willingness to provide abortion care

The success of any role expansion strategy is likely to be influenced by health workers’ willingness to take on the new tasks expected of them. Health workers who are reluctant to perform these tasks may either refuse to deliver them or may deliver poor quality care to their clients. Our synthesis suggests that health workers’ willingness to provide abortion services varied, and was influenced by a number of factors, including their personal views and beliefs about abortion, the stage of pregnancy and method of abortion, and their perceptions of their professional roles.

#### Health workers’ willingness influenced by their personal views about abortion

In all five countries, health workers’ personal views and beliefs about abortion influenced their willingness to provide abortion care services.

In Nepal, specialist and non-specialist doctors, nurses and auxiliary nurse midwives were reported as being generally supportive of the provision of safe abortion services [16, 17], seeing these services as an important contribution to women’s health and perceiving their participation in the provision of these services as a way of helping women in need [18, 19]. Some of them saw a need to increase access to safe abortion services and were concerned about continued unsafe abortion practices [18, 19]. In South Africa, some doctors, nurses and midwives echoed these sentiments, referring to their own prior exposure to the consequences of unsafe abortion, either professionally or in their personal lives [20, 21]; to the consequences of raising children in difficult socioeconomic circumstances [21]; and to their support of the woman’s right to choose [20, 21].

While South African health workers often supported the provision of abortion care, reluctance to provide abortion care because of moral or religious beliefs was also commonly reported among these groups [21–27]. Similar resistance was reported, although to a lesser extent, among specialist doctors in Uruguay [28]; among midwives, specialist and non-specialist doctors, nurses and health officers in Ethiopia [29, 30]; and among health workers in Bangladesh [31–35]. Ethiopian health workers in some reports agreed that unsafe abortion was a serious problem and that there was a need for safe abortion services, but their willingness to provide these services varied [29, 30, 36]. Similarly, doctors in one report from Bangladesh supported the use of abortion care services as a way of achieving the government’s population control objectives, but preferred not to perform these services themselves [37].

While health workers’ personal views towards abortion were mixed, reports from all five countries indicated that health workers were likely to regard some reasons for seeking abortion as more legitimate than others. For instance, some health workers, including nurses, midwives and doctors, found abortion more acceptable if it was due to rape, incest, foetal abnormalities, serious illness or economic hardship [21, 36, 38–41]. Conversely, health workers in some settings were reported as being less sympathetic and more judgmental towards young, unmarried girls seeking abortion [18, 33, 42]. In one Nepalese report, doctors and nurses supported the ban on sex-selective abortions, but acknowledged the pressure women were under to have male children and were concerned that women would seek illegal abortions if turned away [19].

#### Health workers’ willingness influenced by the method of abortion and stage of pregnancy

Health workers’ experiences of and willingness to provide abortion care services were also influenced by the method of abortion and the stage of pregnancy. While health worker perceptions about the effectiveness of medical abortion compared to other methods sometimes varied, nurses and other health workers in reports from South Africa, Ethiopia and Nepal regarded it as simpler to perform [43–45]. Some also found medical abortion more acceptable because they saw it as requiring less of an active role by the provider in managing the abortion process [12, 46].

In South Africa, Ethiopia and Nepal, some specialist and non-specialist doctors, facility managers and other health workers were reported as feeling uncomfortable with the provision of second-trimester abortions [21, 47, 48], often because they found it traumatic to deal with the foetus [12, 21, 24]. In one South African report, midwives involved in second-trimester medical abortions described feeling emotionally unprepared and alone as they were often left unsupported after the doctor had prescribed the drug [39]. These midwives described not wanting to be alone with the woman and the foetus, and appreciated company, even if that person did nothing other than provide moral support [39]. In Ethiopia, midwives or nurses taking care of the expelled foetus and the mother were typically accompanied by another health worker or other member of staff, including cleaning staff [12]. Based on challenges with the implementation of second-trimester abortion services experienced in other countries, programme planners in Nepal intentionally delayed the introduction of second-trimester abortion services, both to ensure that providers had good first-trimester abortion skills, and to prepare providers and garner support for second-trimester services, for instance through values clarification workshops (see below) [6, 47, 49]. They also used staff rotation to avoid burnout because of the emotional burden related to second-trimester abortions [47].

#### Health workers’ willingness influenced by their perceptions of their professional roles

Health workers’ willingness to provide abortion care also appeared to be influenced by their perceptions of their professional roles. In some South African reports, nurses described what they perceived to be a conflict between their pledge to preserve life and their identity as carers of mothers and children on the one hand, and their involvement with abortion on the other [27, 38, 39]. In one of these reports, nurses complained that deliveries and abortions were conducted in hospital units directly opposite each other, which served to emphasise this conflict, and nurses believed that abortion should be performed in a separate clinic [27]. However, in Ethiopia, one key informant described midwives as having become more supportive of the provision of abortion care over time, suggesting that the incorporation of safe abortion services into midwives’ pre-service training led midwives to regard abortion care as an inherent part of their role as health providers [50]. One report in South Africa also found that some health workers, including midwives and nurses, viewed the provision of abortion as part of a natural career trajectory, and as an opportunity to broaden their skills base [21]. Similarly, in Nepal, after some initial concerns, auxiliary nurse midwives who were trained to provide medical abortions expressed confidence in their skills, and a desire to further broaden their skills in abortion care [11].

#### Health workers’ willingness influenced through values clarification workshops

In all five countries, efforts were made to increase health workers’ willingness to provide abortion care services through educational activities. Workshops were held in Uruguay aiming to build commitment among health workers and administrative personnel [51]. In South Africa, Nepal, Ethiopia and Bangladesh, values clarification workshops were used to garner support for abortion services in general and for second-trimester services in particular [12, 32, 49, 50, 52, 53]. These workshops aimed to educate participants about current abortion legislation; allow them to clarify their values and attitudes; encourage change in their attitudes and behaviour towards women seeking an abortion; and ultimately achieve support for the provision of abortion services [20, 21, 26, 49]. Participants suggested that these workshops had been useful in giving them a better understanding of abortion [23] and helped them to acknowledge clients’ rights and needs [47]; assisting those opposed to abortion in “viewing things differently” [21], and helping them to feel comfortable when talking about abortion [12]. However, one report from South Africa pointed out that these workshops were not mandatory and there seemed to be little done to encourage attendance [21].

#### Health workers’ access to, knowledge about, and use of conscientious objection

Health workers seemed to use conscientious objection to opt out of providing abortion care but were not always aware of the limits of the provisions of the law or their legal obligations to ensure that women were not denied care. In South Africa and Uruguay, legislation was in place that allowed health workers to conscientiously object to performing an abortion [5, 8, 54]. However, health workers did not always understand or follow this legislation correctly and guidelines or systems were not always in place to manage the implementation of conscientious objection [5, 8]. In South Africa, health workers in two reports refused to assist in any part of the abortion procedure or provide basic nursing care to abortion clients, citing religious or moral reasons, although they were not entitled to conscientiously object to performing these tasks [21, 27]. In Nepal, Ethiopia and Bangladesh, there was no formal policy for conscientious objection. However, in Nepal, no action was taken towards Nepalese providers who refused to provide abortions based on their conscience [52]. In one report on Ethiopian midwives, most respondents believed that there would be no repercussions for midwives refusing to provide abortion services [30]. In Bangladesh, providers who were unwilling to provide the service were encouraged to refer women elsewhere [32].

### Health workers’ knowledge about abortion legislation and services

Health workers taking on new tasks also need to be knowledgeable about which services are available, to whom, and under what circumstances. However, health worker knowledge about abortion legislation and services varied across settings. In Uruguay, informants suggested that there was widespread knowledge about the law among all health workers, both because of access to training [54] and because of the high-profile legal battles about conscientious objection and denial of care to women due to health system delays that push them beyond the pregnancy stage at which legal abortions are allowed [28]. In South Africa, Ethiopia, Nepal and Bangladesh, on the other hand, knowledge about legislation and available abortion services among health workers appeared to vary but was often described as lacking, both among health workers who provided abortion care and those who did not [9, 18, 21, 29, 30, 32, 36, 40, 49, 55–58]. This apparent lack of awareness had consequences for women’s access to services. For instance, in Bangladesh, some health workers were unaware that second-trimester abortions were legal in certain circumstances and therefore refused to perform the service [32] while some Nepalese health workers wrongly believed that women needed their husbands’ permission to obtain an abortion [18]. In Nepal, particular efforts were therefore made in training programmes for auxiliary nurse midwives to ensure that they would provide services to all women, including young, unmarried women [59].

### Managers’ and co-workers’ attitudes towards role expansion

Role expansion strategies also affect co-workers, including those responsible for managing or supervising the health workers that have been given new tasks, and those who have previously delivered the services in question. Managers’ and co-workers’ views on non-physician provider role expansion varied within and across settings. In Nepal, Bangladesh and Ethiopia, specialist and non-specialist doctors, health officers and others were reported as being positive towards the use of non-physician providers to provide abortion services [37, 60–63]. In Bangladesh, one report suggests that doctors’ own reluctance to provide abortions, either on religious grounds or because they felt that menstrual regulation was “medically unsophisticated”, may have contributed to their willingness to allow non-physician providers to take on this task [37]. Facility managers in one Nepalese report were also positive towards the use of nurses in the provision of first trimester abortion care services, indicating that this could improve continuity of care, decrease the burden on doctors, increase retention of nurses and increase patient satisfaction with health services [64].

However, in the same Nepalese study, there were also some concerns about nurses’ inability to manage severe complications [64]. In reports from Bangladesh and Ethiopia, specialist and non-specialist doctors, nurses and health officers also had some concerns about the safety and effectiveness of using non-physician providers and the lack of good referral systems [37, 63]. In Uruguay, one informant referred to “turf protection” where specialist doctors were reluctant to delegate tasks, such as prescribing abortion medication, to other health workers because they did not feel that these health workers were qualified to provide the service [28]. However, this informant also pointed out that in Uruguay, which is a highly urban country with a high physician to population ratio, few tasks are shared with non-physicians [28]).

In some studies, non-physician providers also experienced resistance from co-workers because of their personal views on abortion. In South Africa, some nurses, midwives, specialist and non-specialist doctors described feelings of rejection, stigma and negative comments because of their work from colleagues who did not provide abortion care [21, 23, 24, 44, 65], particularly when delivering second-trimester abortions [24]. In Bangladesh, some menstrual regulation providers were reported as feeling demoralised by the discrimination and abuse they received from colleagues [32].

#### Women’s attitudes to and experiences of different types of health workers

The success of role expansion strategies may also be influenced by how service users perceive the use of new types of health workers. Several reports noted that women in the five countries were generally satisfied with abortion services and abortion service providers [11, 28, 66–69], although there were also complaints of poor treatment of women [27, 28, 43, 44, 69, 70]. However, women’s attitudes and experiences towards service providers tended not to focus on the category of health worker and his or her level of training. Instead, women primarily referred to issues such as knowing and trusting the health worker, kindness and caring, being able to speak the same language, confidentiality and privacy, cost and closeness to home [33, 63, 67–69, 71–73]. In at least one report, female health workers were also preferred over male health workers [74]. In Bangladesh, the presence or absence of these factors led some women to prefer informal providers [33, 71, 72], while in Nepal and Ethiopia, lay health workers were sometimes preferred for similar reasons [63, 75].

### Health system factors

The success of role extension strategies is also likely to depend on the ability of the health system to adapt to the organisational implications of these strategies. Reports from the five countries suggest that the successful use of non-physician providers could be influenced by health worker workloads and incentives, training and supervision, supply chains and referral systems, and monitoring and evaluation systems.

#### Health worker workloads and incentives

The inclusion of abortion services often had an impact on non-physician providers’ workload. In Ethiopia, midwives who were asked to take on abortion services complained, particularly in the beginning of the programme, that they were being burdened with the doctors’ tasks [12, 50, 76]. In South Africa, one report described how an increase in access to abortions had not been matched with an increase in healthcare facility staff [77], and increased workloads were referred to as one reason for not taking on abortion services among South African nurses [38, 44]. In Uruguay, facilities hired new staff to come in line with the new abortion legislation or redistributed staff where conscientious objection impeded service provision, but a lag between the change in legislation and facilities’ readiness to provide services was initially reported [28].

A related complaint was that increases in health worker workloads were not always reflected in health worker incentives. In South Africa, nurses and midwives indicated that they would be more willing to provide abortion services if they were to receive additional incentives, and expressed frustration that their additional training and certification had not led to an increase in pay [46].

#### Health worker training

The inclusion of abortion care services also had implications for health worker training. The extent to which abortion care had become part of the curriculum in medical, nursing or midwifery schools varied across countries [6, 19, 22, 48, 50, 54, 78]. In Bangladesh, pre-service training in some abortion care services was offered to family welfare visitors, nurses, nurse-midwives and midwives, but pre-service training institutions were reported to suffer from a lack of skilled teachers and teaching tools and few opportunities for hands-on clinical training [78].

The reports suggest that most attention was paid to in-service training. For instance, in South Africa and Nepal, a cascade model or “Training of trainers” approach was used to train abortion providers as efficiently and rapidly as possible [2, 6, 79, 80]. However, the implementation of in-service training posed a number of challenges. In Nepal, healthcare facilities that were used as training centres often found the dual demands of training and regular service provision too demanding [6, 79]. In Ethiopia, a pilot report that aimed to train lay health workers to deliver medical abortions at health posts was made difficult because of an insufficient number of cases at this level of care [68]. In Nepal, South Africa and Ethiopia, nurses, midwives and other health workers working in facilities with staff shortages reported difficulties getting work release to attend training [6, 21, 44, 68], while community-based lay health workers in Nepal found it difficult to attend the few training opportunities they were given due to other commitments [75]. South African abortion providers were also reported as avoiding abortion training because of stigma from colleagues [21]. In South Africa, Nepal, Ethiopia and Bangladesh, the government collaborated with the private sector and with NGOs to increase capacity and provide training in abortion care [2, 12, 21, 32, 33, 57, 79]. However, one report in Bangladesh described variations and inconsistencies in training length and content between the different NGO training programmes and some policy makers called for a common training curriculum [33].

#### Health workers’ access to supportive supervision

Access to supportive supervision, including emotional support from colleagues was acknowledged as important for abortion service providers, but was sometimes reported as lacking [35, 64, 81, 82]. In one report in Bangladesh, supervisors in the public sector were said to struggle with a lack of skills, tools and checklists, although NGOs were described as having strong supervisory procedures [33]. In Ethiopia, partner NGOs were also reported as providing regular supportive supervision, at least in certain areas of the country [50], where a standard checklist was used, feedback was given on the spot and support was provided [12]. However, the long-term sustainability of support from NGOs was reported as a concern [12]. One report from Nepal described how the government worked with a partner NGO to establish long-term support systems for auxiliary nurse midwives by developing teams of local stakeholders whose role it was to maintain regular contact and offer support to these providers [59].

In addition to managerial supervision and support, the reports referred to the importance of support in dealing with the emotional challenges of the work [23, 27, 83, 84]. In one Nepalese report, nurses described feeling supported by managers, doctors and other nurses [64] while in some South African reports, informal support was sometimes available, either from other abortion providers or non-providers [40, 65]. However, the South African reports in particular highlighted a lack of psychological support [23] and a lack of support from managers [39, 40, 65] doctors and the authorities [77]. Support was called for not just at regular intervals, but immediately after a difficult emotional experience [65], and health workers suggested that this could come from colleagues, managers, psychologists or priests [23]. Different solutions were used to offer such support. In Ethiopia, abortion providers from different healthcare facilities were linked to each other so that they could share their experiences and support each other [12]. In South Africa, formal support, for instance through support groups and debriefing sessions, was sometimes offered, particularly in the initial stages of the abortion programme [46]. Health workers also appreciated informal emotional support from colleagues. For instance, working together with a colleague during difficult abortion procedures was described as minimising feelings of loneliness while sharing experiences with colleagues provided an outlet for their emotions [39].

#### Health workers’ access to supplies and referral systems

While the adoption of abortion care services by non-physician providers allows abortion care services to be offered at a broader range of facilities, this requires that these providers have good access to supplies and referral systems. In Nepal, Bangladesh and Ethiopia, poorly equipped facilities and poor access to supplies and drugs were sometimes reported as a problem, particularly for abortion care providers working at primary level and in peripheral areas [6, 9, 33, 50, 75, 85]. In Nepal, while poor supply chain management was one reason for this, the USA’s Helms Amendment, which limits the use of US foreign assistance for abortion, also presented challenges for abortion supply logistics [6]. In reports from Bangladesh, Ethiopia and Nepal, referring women on for further abortion care also had its challenges, sometimes reflecting general weaknesses in the country’s referral systems, including a lack of health workers to refer on to [31, 50, 86]. In one report, Nepalese lay health workers offering pregnancy tests to women were expected to refer women on for abortion services or antenatal care but found referral cards difficult to use and instead accompanied women to health facility appointments [75].

#### Monitoring and evaluation of health workers

Monitoring and evaluation may be particularly important after new tasks have been introduced, and different approaches were used to monitor and evaluate health worker performance and encourage service improvement. In Uruguay, a monitoring and evaluation tool was applied once every 6 months. This involved observations of counselling sessions and an interview with the client followed by meetings with the health worker teams to explain the findings and help to improve the service [54]. In Ethiopia, annual review meetings were conducted where health managers, health workers and facility heads were given the opportunity to discuss their successes and challenges and come up with action points [12]. In Nepal, managers at abortion facilities were trained to use performance improvement checklists to help them identify problems, for instance with regard to staff skills and motivation and facility supplies and functioning, and to develop action plans to address these issues [6]. However, obtaining accurate monitoring data could be challenging as record keeping was often poor, especially when staff were overburdened or where they rotated between different departments [6, 79]. For the Nepalese lay health workers, monitoring and evaluation was further complicated by low literacy levels, although self-reporting of activities during monthly meetings has been one solution to this problem [75]. Another challenge in Nepal was the fact that private facilities had no reporting obligations, making their monitoring data unavailable to the government [6]. Technical and logistical problems and lack of training were also reported in the monitoring and evaluation of menstrual regulation services in Bangladesh [32, 33].

## Discussion

By increasing the types of health worker that can deliver services such as abortion care, services can be delivered by more health workers and at a wider range of facilities, thereby bringing services closer to the people who need them and giving more people access to these services. Once they have been adequately trained, health workers in primary or community care may also be particularly well suited to deliver these services because of closer links to the communities they serve, for instance through better language skills and a better understanding of their clients [87], and because more people have access to these health workers.

Many of the challenges of role expansion in abortion care are similar to other types of health care services. Our synthesis highlights challenges that have been documented for health worker role expansion in abortion care in other countries [88], as well as for other areas of healthcare, including maternal and child health and HIV [89–93]. These include concerns about access to supplies and functioning referral systems when tasks are moved to new levels of facilities [89, 90], problems in ensuring that health workers taking on new roles are sufficiently trained and supervised [89–93], and implications for health worker workloads and salaries [89–93]. However, these challenges reflect the broader weaknesses of many healthcare systems, and are likely to require increased resources and changes to these systems at a far wider level than for abortion programmes alone.

Our synthesis also highlights issues that may be unique to or particularly relevant for abortion services. In contrast to many other health issues, abortion is a topic of debate in most settings. People’s attitudes towards abortion are likely to be influenced by their moral or religious beliefs, their views and experiences regarding woman’s roles and women’s rights, and their views of the rights of women and the foetus [94]. As members of society, health workers are also part of this discourse. In addition, health workers’ attitudes may be influenced by what they regard to be their professional roles; their experiences of stigma and support, of women seeking abortions, and of the procedure itself. Together, these factors may influence health workers’ willingness to provide abortion care services or to support colleagues carrying out these services, as demonstrated here and in evidence from other countries [88].

As is the case for other types of health worker role expansion, it is essential that health workers taking on abortion care services are properly trained to ensure that services are technically sound. The use of in-service training is particularly relevant when new tasks are introduced or standards updated. However, our synthesis shows that in-service training can be difficult to implement. In addition, the effects of most kinds of in-service training and other implementation strategies on quality of care are generally modest [95]. The incorporation of abortion care services into pre-service curricula appears to have been given less attention, but may be an important training opportunity. In addition, pre-service training may help shape health workers’ perceptions of their professional roles [96].

For abortion programmes to have access to enough health workers, and for women to be treated with respect and compassion, health workers also need to be willing and motivated. Technical training alone is therefore unlikely to be sufficient. Our findings suggest that training should also provide abortion care providers with a thorough understanding of the circumstances that may lead women to seek an abortion and an understanding of women’s legal rights. The use of values clarification workshops may be one way of addressing this issue [49]. However, it may be important to involve all facility staff, including non-clinical staff, and to encourage participation or make participation mandatory.

While different types of training can potentially influence health workers’ willingness to provide abortion care, this willingness may be tested by challenges in their everyday work. Our synthesis and other studies suggest a number of ways in which abortion care providers’ working conditions could be improved. One suggestion made by nurses in our synthesis was that abortion services should be provided in separate facilities from labour wards [27]. However, it has also been suggested that women may prefer the increased anonymity that general facilities can offer [97]. In addition, it has been argued that a separation of abortion services from mainstream healthcare services further marginalises abortion and abortion providers [98]. The effects of these and other strategies for improving the working conditions of health workers delivering abortion care need to be evaluated, and should consider the impacts on both women and health workers. Other strategies referred to in our synthesis include the use of health worker rotation; access to emotional support from colleagues or supervisors; and ensuring that health workers carrying out second-trimester abortions are not expected to do this alone. The importance of this type of support has also been referred to by nurses in other studies [99–101].

Despite their best efforts, programme managers may still find that health workers are unwilling to participate in abortion care. Opportunities to opt out varied across the five countries, and the advantages and disadvantages of formal procedures for conscientious objection compared to more informal solutions is unclear from the data. The WHO guidelines on health worker roles in abortion care recommend that “(c)onscientious objection, where allowed, should be regulated, and provision of alternate care for the woman provided” [1]. Our synthesis suggests that health workers in some settings need to be better informed about their options and that formal systems may need better enforcement.

### Study strengths and limitations

Many of the studies we identified focused on the delivery of abortion care services by both physicians and non-physician providers, and did not focus on role expansion specifically. We therefore had to make some assumptions about how factors affecting the implementation of abortion care service delivery by both physicians and non-physicians would apply to role expansion strategies among non-physician providers.

Another challenge was the fragmented nature of the data. Many of the reports we identified offered only snapshots from different parts of each country or at different stages in each programme’s history. This made it difficult to create a coherent understanding of the programmes and to identify processes within programmes over time. For instance, we were not able to gain a good overview of factors that were present when the programmes were introduced and the extent to which these factors changed as the programmes matured. In general, the reports also aimed to answer different questions than our own, with few of the reports focusing on role expansion to non-physician providers specifically. Both of these challenges have been noted before in syntheses of this kind [90].

Another challenge was that the included documents offered varying levels of detail regarding how the data had been collected and analysed and were based on a range of different study designs. This variation implied that it was not possible to apply standardised assessment tools to appraise the quality of each documentation source.

The amount of data that we were able to identify for each country also varied. Reports from Uruguay were particularly limited, which may have reflected the relatively short period of time since the programme was established but may also be a result of our search strategies, which may have biased us towards English language sources of information.

We were, however, able to identify a relatively broad set of materials for the programmes, including programme evaluations and academic studies conducted at different points in the programme lifespan and we supplemented these with feedback from key informants. We also attempted to ensure that these key informants reviewed our final analysis. This allowed for the triangulation of sources, methods and timeframes, and strengthened the validity of our findings.

## Conclusions

The World Health Organization recommends a range of strategies to expand health worker roles in the delivery of abortion and post abortion care services [1]. To increase the likelihood of success for these strategies, programme planners need to consider how they can build willingness and motivation among health workers, and create working conditions that ensure the provision of high-quality and compassionate services to the women who need them.

## Addition files

Additional file 1: Search strategy. (DOCX 14 kb)
Additional file 2: Documents that contributed to the findings reported in this paper. (DOCX 29 kb)
