# Supplementary material for: Implementation considerations when expanding health worker roles to include safe abortion care: a five-country case study synthesis
Source: BMC Public Health. 2017 Sep 21;17:730. doi: 10.1186/s12889-017-4764-z (PMC5609023; doi:10.1186/s12889-017-4764-z)
Supplement: Additional file 1: — Search strategy. (DOCX 14 kb) [file 12889_2017_4764_MOESM1_ESM.docx]

**Additional file 1. Search strategy**

| *Ovid MEDLINE In-Process & Other Non-Indexed Citations, Ovid MEDLINE Daily, Ovid MEDLINE and Ovid OLDMEDLINE 1946 to present – Searched 25 May 2014* |
| --- |
| 1. (abortion* or (pregnanc* and terminat*) or miscarriage* or unintended pregnanc* or unwanted pregnanc* or abortifacient or ((menstruat* or menstrual) and regulat*) or ((delayed or suspended) and menstruat*)).tw. or (menstruation/ and (delayed or suspended or regulat*).tw.) |
| 2. exp Abortifacient Agents/ or Vacuum Curettage/ |
| 3. abortion, induced/ or abortion, eugenic/ or abortion, legal/ or abortion, therapeutic/ or pregnancy reduction, multifetal/ |
| 4. abortion, spontaneous/ or abortion, habitual/ or abortion, incomplete/ or abortion, missed/ or abortion, septic/ or abortion, threatened/ or embryo loss/ |
| 5. or/1-4 |
| 6. midwifery/ or nurses / or nurse clinicians/ or nurse midwives/ or nurse practitioners/ or family nurse practitioners/ or nurses, community health/ or nurses, international/ or nurses, male/ or nurses, public health/ or nursing staff/ or nurses aides/ or students, nursing/ |
| 7. allied health personnel/ or community health workers/ or emergency medical technicians/ or home health aides/ or operating room technicians/ or pharmacists' aides/ or physician assistants/ or caregivers/ or hospital auxiliaries/ or pharmacists/ |
| 8. Health Services, Indigenous/ or Medicine, Traditional/ or Integrative Medicine/ or Complementary Therapies/ or Herbal Medicine/ or Chiropractic/ |
| 9. medicine, traditional/ or medicine, african traditional/ or exp medicine, arabic/ or medicine, ayurvedic/ or exp medicine, east asian traditional/ or shamanism/ |
| 10. exp complementary therapies/ or acupuncture therapy/ or herbal medicine/ or chiropractic/ |
| 11. (nurse* or nursing auxiliar* or nursing assistant* or midwife* or midwives).tw. |
| 12. (paraprofessional* or paramedic* or paramedical or allied health personnel or allied health worker* or support worker* or home health aide* or trained volunteer* or ((trained or lay or community or village or maternal or rural) adj3 (health volunteer* or health worker* or healthcare worker* or health care worker* or nutrition worker* or health agent* or health guide* or health visitor* or health advocate* or health promoter*)) or treatment supporter* or TBA* or shasthyo sebika or agente communitario de saude or visitador* or women group leader* or accompagnateur* or saksham sahaya or anganwadi worker* or behvarz or brigadistas or lady health worker* or trained mother* or community drug distributor* or (lay adj (volunteer* or worker* or visitor* or attendant* or aide* or support* or person* or helper* or caregiver* or consultant* or assistant* or staff))).tw. |
| 13. (clinical officer* or physician assistant* or medical assistant* or clinical associate* or health officer* or non-physician clinician* or non-professional clinician* or surgical technician* or nonclinician* or non-specialist doctor* or medical technician* or medical licentiate practitioner* or assistant medical officer* or non-clinician* or non-specialist doctor*).tw. |
| 14. ((indigenous or traditional or integrative or complementary or herbal or ayurved* or homeopath* or acupunctur* or herbal* or folk or chinese or african or korean or mongolian or tibetan or asian or eastern or oriental or ayush or alternative or naturopath* or siddha or tuina or unani or osteopath*) adj (medicine or therap* or healer* or healing or practitioner* or provider*)).tw. |
| 15. (pharmacy or pharmacies or pharmacist* or (dispensary adj (assistant* or technician* or intern*)) or chemist or chemists or ((community or service*) adj3 pharmaceutical)).tw. |
| 16. (task shifting or task-shifting or taskshifting or task sharing or task-sharing or tasksharing).tw. |
| 17. or/6-16 |
| 18. 5 and 17 |
| 19. (nepal* or bangladesh* or south africa* or ethiopia* or uruguay*).ti,ab,cp. |
| 20. ethiopia/ or south africa/ or uruguay/ or bangladesh/ or nepal/ |
| 21. 19 or 20 22. 18 and 21 |
| *Search strategy was adapted for Global Health, CINAHL (EBSCO), Popline and the WHO Global Health Library – Searched 25 May 2014. The search was repeated 7^th^ November 2016.* |
