# Supplementary material for: Implementation considerations when expanding health worker roles to include safe abortion care: a five-country case study synthesis
Source: BMC Public Health. 2017 Sep 21;17:730. doi: 10.1186/s12889-017-4764-z (PMC5609023; doi:10.1186/s12889-017-4764-z)
Supplement: Additional file 2: — Documents that contributed to the findings reported in this paper. (DOCX 29 kb) [file 12889_2017_4764_MOESM2_ESM.docx]

## Additional file 2. Overview of documents that contributed to the synthesis findings

| **Reference** | **Aim of the document** | **Source of data** |
| --- | --- | --- |
| Bangladesh | | |
| 1. Amin R, 1988 | “This article evaluates the performance of three urban menstrual regulation training and service programs in Bangladesh” | Quantitative survey of menstrual regulation providers, physicians and paramedical workers |
| 1. Akhter HH, 2001 | Description of the use of midlevel providers in the delivery of menstrual regulation in Bangladesh | Descriptive data |
| 1. Akhter HH, 2001b | Description of the menstrual regulation programme in Bangladesh | Literature review, the personal experience of the author as the first menstrual regulation service provider, and interviews with senior government managers and an NGO executive |
| 1. Caldwell 1999 | “In (this study, we examined) the factors influencing (women’s) decision to end their pregnancy, how the decision was made, any distinct health-seeking behaviours, the provider chosen and the consequences of undergoing a termination” | Structured interviews with married women |
| 1. Chowdhury SNM, 2004 | “This paper reports a situation analysis of the MR Programme under the [Health and Population Sector Programme] (HPSP)” | The World Health Organization rapid evaluation methodology – including a planning workshop with stakeholders, in-depth interviews and group discussions with programme managers, service providers (cadre not specified), and men and women visiting healthcare facilities, and observations at healthcare facilities. |
| 1. Das T, 2014, 2016, 2017 | To gather information about the use of different healthcare workers to provide abortion services in the public sector in Bangladesh, and to gather feedback on the study findings. | Personal communication: key informant feedback on manuscript |
| 1. Hena IA, 2013 | “This operations research tested the feasibility of introducing MR with medication (MRM) in Bangladesh and assessed accessibility of the combination regime of Mifepristone and Misoprostol in urban and rural health facilities” | Semi-structured interviews with service providers (cadre not specified) and clients, observation of client-provider interactions, review of case records and service statistics |
| 1. Hossain A, 2008 | Description of the menstrual regulation programme in Bangladesh | Descriptive data |
| 1. IPAS, 2013 | To describe an assessment of the specific needs and gaps in pre-service education for midlevel providers, and to identify opportunities, strategies and recommendations for creating more equitable access to safe abortion care. | Curriculum review; government documents; key informant interview; assessment of midlevel provider performance and training; and site observation visits. |
| 1. Mahmud I, 2015 | “The aim of this study was to understand the context in which the diverse group of close-to-community sexual and reproductive healthcare providers operates in Bangladesh.” | In-depth interviews with menstrual regulation clients, focus group discussions with married men and women in communities, and semi-structured interviews with formal and informal providers (cadre not specified). |
| 1. Marlow HM, 2016 | “In this study, we sought to understand women’s experiences using medication for menstrual regulation in rural and urban Bangladesh” | In-depth interviews with women who had used medication for menstrual regulation |
| 1. Nashid T, 2007 | “This study (…) was designed with the aim of exploring and describing the perceptions of a sample of women in Dhaka about (menstrual regulation)” | Semi-structured interviews with married women |
| 1. Rosenberg MJ, 1981 | “In this study, we surveyed the attitudes toward abortion of physicians in rural *thana* health complexes and subdivision and district hospitals” | Quantitative survey of physicians |
| 1. Ross GC, 2002 | “This study was conducted to determine the factors that influenced the development of and support for the menstrual regulation policy in Bangladesh, in order to explore how far those factors might influence future sustainability” | Focused and open-ended interviews with representatives from “all important stakeholder groups”, including representatives from government and from non-government organisations. |
| 1. Sultana S, 2014, 2016, 2017 | To gather information about the use of different healthcare workers to provide abortion services in the public sector in Bangladesh, and to gather feedback on the study findings. | Personal communication: key informant feedback on manuscript |
| 1. Vlassoff M, 2012 | “This report provides new information on factors associated with access to and quality of MR and PAC services in Bangladesh” | Survey of public and private sector healthcare facilities; survey of professionals, including ob-gyns, non-specialist physicians, programme managers, administrative health officials, and other health professionals; service statistics |
| **Reference** | **Aim of the document** | **Source of data** |
| Ethiopia | | |
| 1. Abdi J, 2011 | “(T)he present study seeks to answer the following key research questions; “what does perceptions on safe abortion look like among health care service providers?”, “what are the factors which affect the perception of health providers towards safe abortion?” | Questionnaire with nurses, midwives, health officers and specialist and non-specialist physicians. |
| 1. Afework, MF, 2015 | To explore the acceptability of involvement of Health Extension Workers in medical abortion by women who seek services and abortion service providers, and to assess willingness and confidence of Health Extension Workers | In-depth interviews with women, diploma nurses, degree nurses, health officers, and Health Extension Workers |
| 1. Alemu FF. 2010 | “The main objective of this research is: to explore the factors influencing minors' access to safe abortion services in Addis Ababa, including the effect of the new abortion law.” | In-depth interviews, focus group discussions, observation with girls who had had abortions and with health professionals (cadre unclear, but appears to include centre managers, physicians, nurses and counsellors). |
| 1. Berhe A, 2014, 2016, 2017 | To gather information about the use of different healthcare workers to provide abortion services in the public sector in Ethiopia, and to gather feedback on the study findings. | Personal communication: key informant feedback on manuscript |
| 1. Leitner Center, 2010 | Description and discussion of the impact of USA’s foreign policy on abortion services in Ethiopia | Descriptive study |
| 1. Desta D, 2014, 2016, 2017 | To gather information about the use of different healthcare workers to provide abortion services in the public sector in Ethiopia, and to gather feedback on the study findings. | Personal communication: key informant feedback on manuscript |
| 1. ESOG, 2014 | “In Ethiopia, the knowledge, belief, attitude and skill in abortion as well as the knowledge and attitude towards the abortion law is not known. Therefore this study is designed to assess this knowledge gap.” | Questionnaire with health workers, including nurses and doctors. |
| 1. Holcombe SJ, 2014, 2016, 2017 | To gather information about the use of different healthcare workers to provide abortion services in the public sector in Ethiopia, and to gather feedback on the study findings. | Personal communication: key informant feedback on manuscript |
| 1. Holcombe SJ, 2015 | “This research aims to analyze the beliefs that might affect implementation of a liberalized law in Ethiopia, and more specifically to examine the readiness of midwives, the frontline providers, to take part in expansion of access to abortion services.” | Survey among midwives and in-depth interviews with midwifery students. |
| 1. Prata NG, 2011 | “The overall goals of this project were to provide empirical evidence to inform the development of  service and clinical guidelines for the provision of CAC services in Ethiopia and to contribute to a  reduction in morbidity and mortality due to unsafe abortion.” | Assessment of patient records, questionnaire with women who had undergone an abortion |
| 1. Technical and Procedural Guidelines for Safe Abortion Services in Ethiopia, 2006 | Guidelines for safe abortion services | Technical and procedural Guidelines |
| 1. Tsegay A, 2011 | “The objectives of the study are to compare the safe abortion care service facts on the guideline with the knowledge, attitude and practice of health practitioners and to know the understanding of the providers to some of the SAC questions used for comparison.” | Questionnaire with health providers, including doctors, nurses and health officers |
| 1. Yirga A, 2009 | “To assess status of safe abortion service utilization and levels of satisfaction in selected public health facilities of SNNPR, Ethiopia, 2008/09.” | Questionnaire and semi-structured in-depth interviews with women seeking abortion care services. |
| **Reference** | **Aim of the document** | **Source of data** |
| Nepal | | |
| 1. Andersen KL, 2016 | “This article describes a training and support strategy to train auxiliary nurse-midwives (ANMs), already certified as skilled birth attendants, as medical abortion providers and expand geographic access to safe abortion care to the community level in Nepal”. | Descriptive program evaluation |
| 1. Andersen K, 2013 | “This evaluation assessed the success of a pilot program that trained female community health volunteers (FCHVs) in early pregnancy detection using urine pregnancy tests (UPTs), counseling, and referral to appropriate antenatal, safe abortion, or family planning services.” | In-depth interviews with lay or community health workers and other reproductive health service providers (cadre not specified). |
| 1. Basnett I, 2012 | “This study evaluates the acceptability and quality of comprehensive abortion care services provided by trained nurses in Nepal.” | Facility logbooks registry, nurse provider interviews, facility assessments, facility manager interviews and procedure observation checklists. Descriptive statistics reported. |
| 1. CREHPA, 2005 | “The objective of this formative needs assessment was to determine what information and communication channels are needed for each of the target audiences in order to develop and implement activities aimed to reduce the social, familial, attitudinal, and knowledge barriers that women face with accessing safe abortion services in Nepal.” | Narrative focus group discussions, figurehead group role plays/ discussions, in-depth interviews, and semi-structured interviews with women who had had abortions and their partners. |
| 1. CREHPA, 2009 | “[T]he End-line survey assessed and documented the project performances in terms of [knowledge, attitude, perceptions about abortion law, safe abortion services and perceived benefits from the program]. | Questionnaire with men and women, paramedics and chemists, law enforcers, and community based health providers. |
| 1. CREHPA, 2009b | “The overall objectives of the endline survey was to assess the effectiveness of the pilot MA program in terms of increasing knowledge and acceptance of MA among clients; examine providers’ perceptions of the IEC materials and orientation training received by them and assess the role of community-based health volunteers and pharmacy shops in increasing demand for MA” | Interviews with MA service providers (including doctors, nurses and community health workers) and clients. Survey with married women of reproductive age. |
| 1. Hyman AG, 2008 | “This paper describes experiences and lessons learned about how to establish safe second trimester abortion services in low-resource settings in the public sector in three countries: Nepal, Viet Nam and South Africa.” | Descriptive study |
| 1. Karki C, 2009 | “This survey was carried out to assess the functioning of existing abortion services in 12 Government approved CAC sites of three districts.” | Direct observation of and semi-structured interviews with doctors and nurses. |
| 1. KC N, 2011 | “To determine the best way to implement the new strategies of medical abortion into the existing health system of Nepal; and to facilitate its full-scale implementation, monitoring and evaluation.” | Baseline study, implementation phase and end line study, with focus on auxiliary nurse midwives and staff nurses. Study used both qualitative and quantitative methods, including facility assessment, key informant interviews (type of informants not specified) and facility service statistics. |
| 1. Moller A, 2012 | «This study investigated the experiences, opinions, and attitudes of the staff about their work at safe abortion service centres in the Kathmandu Valley and identified areas in which the health care staff stated the need for improvement”. | Qualitative, semi-structured interviews with doctors and nurses |
| 1. Puri M, 2012 | “This paper examines health care workers’ views of abortion legalization, and changes that they have observed in their practices.” | In-depth interviews with gynocologists/physicians, nurses, counsellors and hospital administrators. |
| 1. Puri M, 2015 | “This paper describes key components of the operations research study, key research findings, and follow-up actions that contributed to create a conducive environment and evidence in scaling up medical abortion services in rural areas of Nepal.” | Semi-structured interviews with auxiliary nurse midwives, and structured interviews with female community health volunteers and with women who received medical abortion carried out as part of an effectiveness study. |
| 1. Samandari G, 2012 | “This paper examines factors that  contributed to rapid, successful implementation of legal abortion in this mountainous republic, including deliberate attention to the key areas of policy, health system capacity, equipment and supplies, and information dissemination.” | Descriptive study |
| 1. Shakya G, 2004 | “This paper describes the development of policy and programme strategies for implementing the new [abortion] law [….).” | Descriptive study |
| 1. Shrestha M, 2011 | To evaluate the successes and areas for improvement in nurse provision of comprehensive abortion care services | Quantitative analysis of data collected through nurse provider interview, procedure observation checklist, facility assessment, facility manager interview, Comprehensive Abortion Care logbook data from facility. |
| 1. Singh M, 2007 | “Objective: to see whether advocacy for abortion law and comprehensive abortion care sites after legalization of abortion in Nepal is adequate among educated people (above school leaving certificate).” | Questionnaire with participants with health backgrounds (including nurses, nursing students and doctors) and non-health backgrounds |
| 1. Tamang A, 2005 | “The study presented in this paper was aimed at understanding the current availability and use of medical abortion in Nepal and exploring what service providers felt about its use for expanding access to safe abortion in the country” | Questionnaire with obstetricians and gynaecologists, general practitioners, paramedics, ayurvedic and homeopathic practitioners, chemists, and executive members of relevant professional associations |
| 1. Tamang A, 2014, 2016, 2017 | To gather information about the use of different healthcare workers to provide abortion services in the public sector in Nepal, and to gather feedback on the study findings. | Personal communication: key informant feedback on manuscript |
| 1. Thapa S, 2010 | “This paper examines the client profile, the context of demand for services, affordability and satisfaction with services”. | Survey among women using abortion services |
| 1. Turner KL, 2008 | “This article explains the basic principles of values clarification and their application in workshops on abortion, particularly second trimester services” | Descriptive study |
| 1. Family Health Division, 2005 | “This booklet documents the early steps and achievements in the process of implementing the reformed abortion law in Nepal, highlighting the roles and activities of the many stakeholders involved, which include government departments, local non-government organisations (NGO), the private sector, and international NGOs (INGO).” | Descriptive study |
| **Reference** | **Aim of the document** | **Source of data** |
| South Africa | | |
| 1. Alblas M, 2014, 2016, 2017 | To gather information about the use of different healthcare workers to provide abortion services in the public sector in South Africa, and to gather feedback on the study findings. | Personal communication: key informant feedback on manuscript |
| 1. Cooper D, 2014, 2017 | To gather information about the use of different healthcare workers to provide abortion services in the public sector in South Africa, and to gather feedback on the study findings. | Personal communication: key informant feedback on manuscript |
| 1. Dickson-Tetteh K, 2002 | “[W]e conducted a comprehensive evaluation of the quality of trained midwives’ abortion care service provision.” | Observation, reviewing records and patient charts, closed-ended interviews with patients and midwives |
| 1. Engelbrecht MC, 2000 | “A study was undertaken to determine the nature and extent of […] impediments to the implementation of the Choice on Termination of Pregnancy Act”. | In-depth, unstructured interviews with two senior officials from the Department of Health; questionnaire with women who had undergone an abortion; questionnaire and interviews with facility managers, doctors, midwives, nurses and counsellors who delivered abortion services or referred women to these services |
| 1. Gabriel M, 2014, 2016, 2017 | To gather information about the use of different healthcare workers to provide abortion services in the public sector in South Africa, and to gather feedback on the study findings. | Personal communication: key informant feedback on manuscript |
| 1. Gmeiner AC, 2000 | To identify, explore and describe nurses’ experience of being directly involved with women who terminate their pregnancy. | Phenomenological interviews with nurses |
| 1. Harries J, 2007 | “This paper reports on results from a qualitative study that explored the factors that contributed towards women accessing abortion services in their second trimester of pregnancy.” | In-depth interviews with women seeking second trimester abortions |
| 1. Harries J, 2009 | “This paper reports on results from a qualitative study that explored knowledge, attitudes and opinions of health service providers who are likely to play a critical role in determining access to and the quality of these services.” | In-depth interviews and one focus group discussion with health care providers, including doctors, nurses and midwives. |
| 1. Harries J, 2012 | “This study set out to explore health service providers’ and health managers’ perceptions and experiences of second trimester abortion services in the Western Cape Province, South Africa, with a special emphasis on service quality and capacity and the potential for service improvement.” | In-depth interviews  with doctors, nurses and managers |
| 1. Harries J, 2014 | “This paper sets out to highlight the ambiguity and inconsistencies regarding the interpretation and implementation of conscientious objection by health care providers and its subsequent impact on abortion service provision.” | In-depth interviews among doctors and nurses, managers and “policy influentials”. |
| 1. Harries J, 2014, 2016, 2017 | To gather information about the use of different healthcare workers to provide abortion services in the public sector in South Africa, and to gather feedback on the study findings. | Personal communication: key informant feedback on manuscript |
| 1. Harrison A, 2000 | “This study explored attitudes and beliefs about abortion and the Termination of Pregnancy Act among primary care nurses and community members in a rural district in order to better understand barriers to implementation of the new law.” | Questionnaire and in-depth interviews with nurses and women in the community. |
| 1. Mamabolo L, 2010 | “(T)o gain insight into the experiences of registered nurses providing [termination of pregnancy] services and to provide relevant recommendations.” | Phenomenological interviews with registered nurses |
| 1. Mayers P, 2005 | “The aim of the study was to explore the lived experience of midwives who assist with terminations of pregnancy in a tertiary level hospital.” | In-depth interviews with registered nurses/midwives |
| 1. Mitchell EMH, 2005 | “..to assess the impact of the workshops at the individual, community and facility levels.” | Survey of workshop participants (including nurses, midwives, managers, social workers) , interviews with workshop facilitators, review of workshop reports and action plans, and a site visit |
| 1. Mokgethi NE, 2006 | “This study attempted to investigate professional nurses’ attitudes towards providing towards rendering termination of pregnancy services at a tertiary hospital in the North West Province of South Africa.” | Questionnaire with professional nurses |
| 1. Orner P, 2010 | To explore HIV-positive women’s abortion decisions | In-depth interviews with HIV-positive women seeking abortion |
| 1. Poggenpoel M, 1998 | “The objectives of this project were to explore and describe nurses’ experience of abortion (…)”. | Focus group interviews, a phenomenological interview, open-ended questionnaires and participant observation with nurses |
| 1. Potgieter C, 2004 | “The objectives of the study were to investigate how TOP [termination if pregnancy] nurses account for their decision to be trained as providers (what motivates nurses to undertake TOP training); (and) compile a profile of the characteristics and/or  beliefs held by nurses who choose to become TOP providers (…)” | Focus group interview and semi-structured interviews with nurses |
| 1. Smit I, 2009 | “This case study explores the situation of certified nurses and the context in which they provide abortion care…” | Questionnaires with women who had had abortions, nurses who provided abortions and nursing students, checklist, and semi-structured interviews with various role players. |
| 1. Trueman KA, 2013 | “I explore the history of the law, subsequent legal challenges, and new threats to women’s access to abortion services, including service delivery issues that may influence the future of public health in the country.» | Descriptive study |
| 1. Turner KL, 2008 | “This article explains the basic principles of values clarification and their application in workshops on abortion, particularly second trimester services” | Descriptive study |
| 1. Varkey S, 2000 | Description and discussion of abortion services in South Africa | Viewpoint, descriptive data |

## Additional file 6. Documents that contributed to the findings reported in this paper (Uruguay)

| **Reference** | **Aim of the document** | **Source of data** |
| --- | --- | --- |
| Uruguay | | |
| 1. Cóppola F. 2013 | To explore the nuances in the legislation on conscientious objection. | Opinion piece |
| 1. Coppola F, 2016 | To analyse the attitudes and behaviour of gynaecologists in Uruguay with respect to the right to conscientious objection that is included in the law concerning voluntary termination of pregnancy” | Document analysis. Focus on gynaecologists |
| 1. Fiol V, 2012 | “To describe the initial stages of the implementation of a risk-reduction model […] to shield women from unsafe abortion in a traditional community on the Uruguayan-Brazil border”. | Questionnaire and in-depth interviews with policy makers, health professionals (cadre unspecified), support staff, pharmacists, informal sellers of misoprostol and women seeking care. |
| 1. Labandera MA, 2014, 2016, 2017 | To gather information about the use of different healthcare workers to provide abortion services in the public sector in Uruguay, and to gather feedback on the study findings. | Personal communication: key informant feedback on manuscript |
| 1. Labandera A, 2016 | To review the history of the creation of the risk and harm reduction model applied to unsafe abortion | Descriptive study |
| 1. Silva M, 2014, 2016, 2017 | To gather information about the use of different healthcare workers to provide abortion services in the public sector in Uruguay, and to gather feedback on the study findings. | Personal communication: key informant feedback on manuscript |
